# ASSOCIATION OF 10-YEAR WALKING TRAJECTORIES WITH COGNITIVE FUNCTION IN OLDER ADULTS: ADULT CHANGES IN THOUGHT STUDY

**DOI:** 10.1093/geroni/igz038.073

**Published:** 2019-11-08

**Authors:** Paul Gardiner, Barbara J Jefferis, KatieRose Richmere, Andrea Z LaCroix, Paul K Crane, Eric B Larson, Dori E Rosenberg

**Affiliations:** 1 The University of Queensland, Brisbane, Queensland, Australia; 2 University College London, London, England, United Kingdom; 3 Kaiser Permanente Washington Health Research Institute, Seattle, Washington, United States; 4 University of California San Diego, La Jolla, California, United States; 5 University of Washington, Seattle, Washington, United States

## Abstract

We identified trajectories of older adults’ walking and their associations with cognitive function. Data on walking (days/week) were collected at baseline of the Adult Changes in Thought study and every two years for 10 years. Cognitive function was assessed by the Cognitive Abilities Screening Instrument (CASI) at year 12. Group-based trajectory analyses identified trajectories among 763 participants (baseline age 70±5 years, 60% female). Regression models, adjusted for baseline sociodemographic and health factors, examined associations with cognitive function. Five walking trajectories were identified: consistently inactive (18.1%), medium active (21.9%), early decline (15.8%), late decline (18.4%), and consistently active (25.8%). Mean CASI score was 92.0 (SD 6.9). CASI scores were lower in early b = -1.66 (95%CI: -2.97, -0.35) and late decline b = -1.89 (-3.26, -0.51) groups, with no difference in consistently active and inactive groups, compared to the medium active trajectory group. Ten-year walking trajectories may determine late-life cognitive function.

